# Gait retraining for runners with patellofemoral pain: A protocol for systematic review and meta-analysis

**DOI:** 10.1097/MD.0000000000025789

**Published:** 2021-05-14

**Authors:** Huan Xiao, Zeng Li, Shoubin Lei

**Affiliations:** Department of Orthopedics, Bijie Traditional Chinese Medicine Hospital, People's Republic of China.

**Keywords:** gait retraining, patellofemoral pain, protocol, systematic review

## Abstract

**Introduction:**

Patellofemoral pain (PFP) is highly prevalent in runners. Physical therapies were proved to be effective in the treatment of PFP. Gait retraining is an important method of physical therapy, but its effectiveness and safety for PFP remained controversial. Previous review suggests gait retraining in the treatment of PFP warrants consideration. However, recent publications of randomized controlled studies and case series studies indicated the positive effect of gait retraining in clinical and functional outcomes, which re-raise the focus of gait retraining. This paper will systematically review the available evidence, assessing the safety and effectiveness for the use of gait retraining for runners with PEP.

**Method and analysis:**

A systematic review of relevant studies in Pubmed, Embase, SCOPUS, and Cochrane Library were synthesized. Inclusion criteria are studies evaluating clinical outcomes (i.e., changes to pain and/or function) following running retraining interventions in symptomatic running populations; Studies with less than 10 participants in total or in the running retraining intervention group were excluded. The primary outcomes measured will be pain score, Lower extremity functional scale and training related injuries or complications. Review Manager (Revman Version 5.3) software will be used for data synthesis, sensitivity analysis, meta regression, subgroup analysis and risk of bias assessment. A funnel plot will be developed to evaluate reporting bias and Begg and Egger tests will be used to assess funnel plot symmetries. We will use the Grading of Recommendations Assessment, Development and Evaluation system to assess the quality of evidence.

**Ethics and dissemination:**

Our aim is to publish this systematic review in a peer-reviewed journal. Our findings will provide information about the safety of gait retraining and their effect on reliving pain and improving function of lower limb on runners with PEP. This review will not require ethical approval as there are no issues about participant privacy.

**Strength and Limitations**

- It is a review that included most recent studies that evaluate gait retraining for runners with PFP.
- The Cochrane Collaboration tool and The Grading of Recommendations Assessment, Development and Evaluation will be used to further evaluate study findings.
- Methodological and clinical heterogeneities will be exit based on the variation of included studies (RCTs and case series) and varied retraining methods (step rate, foot strike, treadmill or ground, training session, time) in included studies
- Pool analysis of available studies for analysis may not allowed to perform. The basic characteristic such as sex, BMI, and running volume may not comparable.

## 1 Introduction

Running is a popular exercise for both recreation and sports.^[1]^ It is proved to be beneficial to cardiac, mental and metabolic health.^[2–4]^ Besides its benefits, running related injuries that reported occurrence rate ranging from 19% to 78% could not be ignored.^[1,5]^ As one of the most common injuries, patellofemoral pain (PFP) was also thought to be the most common overall incidence of musculoskeletal pain amongst recreational runners.^[1,6]^ PFP is defined as pain around or behind the patella aggravated by load the patellofemoral joint activities.^[7]^ Some biomechanical studies found that hip adduction strength,^[9]^ internal rotation, rearfoot eversion,^[8,9]^ and running technique^[10,11]^ that could affect patellofemoral joint load have been related to PFP. Multiple interventions have been developed in an attempt to prevent the occurrence of PFP and release symptoms of PFP runners, including education, local muscle strengthening, and running with foot orthoses et al. However, a lack of effective long-term treatment strategies remains a source of frustration for many runners and clinicians.^[12]^

Gait retraining was defined as the implementation of any cue or strategy to alter an individual's running technique.^[13]^ It was introduced to treat lower limb injuries in runners by reducing load in certain muscle groups and joints.^[14]^ Common gait retraining included altering step rate, strike pattern, hip and knee motion, trunk position, step width, and impact loading variables. Biomechanical and clinical studies have reported the positive effect of gait retraining in kinematics, kinetics and clinical outcome.^[15–17]^ A previous systematic review has synthesis available evidence with international expert opinions on the use of gait retraining, and they indicated it warrants consideration when treating lower limb injuries and limited evidence of gait retraining in the treatment of PFP was were not available to give a further estimate.^[12]^ Recently, some studies of gait retraining focus on clinical practice and comparison with other rehabilitations intervention exit,^[18–21]^ however, the results were controversial. Therefore, an objective and systematic examination on the efficacy and safety of gait retraining for PFP is needed. In our research, we planned to conduct a systematic review and if available, performed a meta-analysis to evaluate the evidence from all available randomized controlled trials (RCTs) that evaluate gait retraining on decreasing PFP.

## 2 Methods

### 2.1 Search strategy

**Figure 1:**
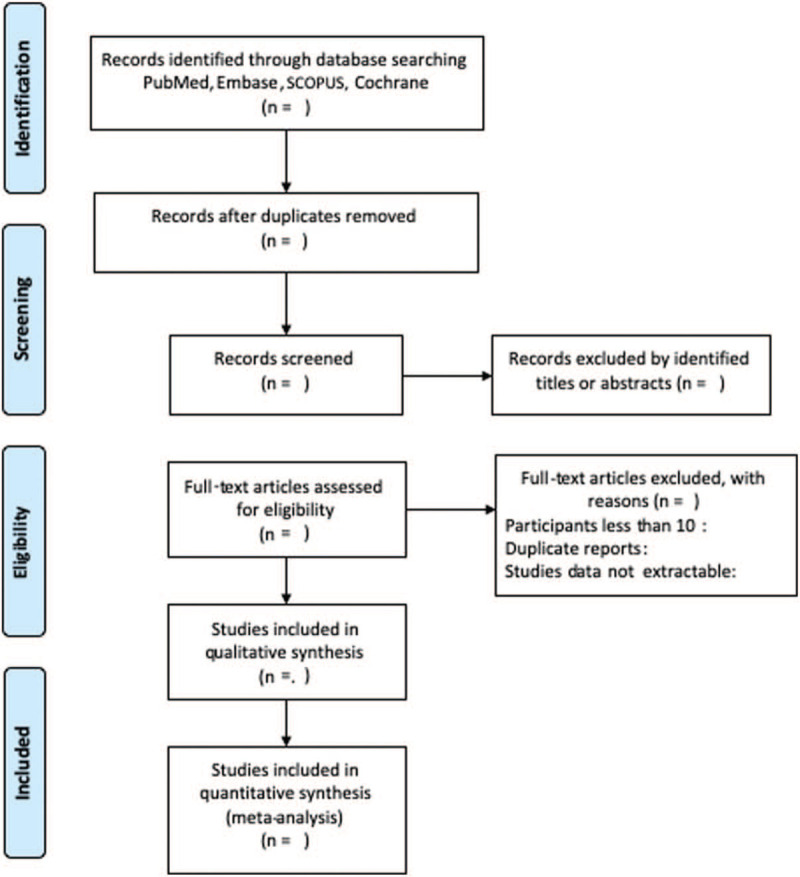
Flow diagram of the relevant study selection process.

We will conduct a systematic review and if available, meta-analysis will be performed to identify relevant studies involving gait retraining and PFP in electronic databases (Fig. 1). Two reviewers independently searched the electronic databases including Pubmed, Embase, SCOPUS, and Cochrane Library up to March 2021 using the following keywords and their combinations: gait retraining, patellofemoral pain. The search strategies in Pubmed were as follow: ((((running) OR runner)) AND (((((((((((gait retraining) OR running technique) OR running method) OR running cadence) OR strike pattern) OR step width) OR step frequency) OR step rate) OR step length) OR stride frequency) OR stride length)) AND (((((Pain Syndrome, Patellofemoral) OR anterior Knee Pain Syndrome) OR Patellofemoral Syndrome) OR Patellofemoral Pain Syndrome) OR Patellofemoral Pain Syndrome[MeSH Terms]).

### 2.2 Inclusion and exclusion criteria

The inclusion criteria will be defined before searching, and the study inclusion eligibility was determined by the following population, intervention, comparator, outcomes, and study design criteria: Studies evaluating running gait retraining or in conjunction with other interventions were considered if the effects of retraining could be clearly delineated (e.g., altering footwear combined with instruction of strike pattern, compared with altering footwear alone). The age of the patients and follow-up periods were not restricted, and the publication language was limited in English. Studies with less than 10 participants in total or in the running retraining intervention group were excluded.

### 2.3 Data extraction and quality assessment

Two investigators will independently extract the relevant data from each study, which included the first authors name, year of publication, country, study design, details of the intervention and control, and the follow-up duration, and outcome measurements for each study. Any uncertainty will be discussed by 2 reviewers and resolved by consensus with discussion with another reviewer. We will contact the corresponding authors of the included RCTs to obtain any missing data when necessary. The Cochrane Collaboration tool^[22]^ will be used to assess the methodological quality and risk of bias of the included studies, including randomization, allocation concealment, blinding method, selective reporting, group similarity at baseline, incomplete outcome data, compliance, timing of outcome assessments, and intention-to-treat analysis. The Grading of Recommendations Assessment, Development and Evaluation approach will be used to evaluate the quality of evidence of the included studies.^[23]^ Reviewers will take into account limitations of the study, inconsistencies, indirect evidence, inaccuracies and publication bias.

### 2.4 Outcome measures

The primary outcome measures that will be evaluated in our review included visual analogue scales to assess knee pain. Knee function was assessed using Lower extremity functional scale, and training related injuries or complications (e.g., calf soreness, ankle injury, and fatigue) to evaluate the safety. The secondary outcomes will be Knee Outcome Survey of the Activities of Daily Living Scale, recurrence rate of PFP and satisfaction of participants.

### 2.5 Statistical analysis and data synthesis

The meta-analyses will be performed using Review Manager (Revman Version 5.3., the Cochrane Collaboration, Oxford, UK). Given the characteristics of the data extracted for the review, continuous outcomes will be expressed as the mean difference (MD) with 95% confidence intervals (CIs). An assumption that the standard deviations (SDs) of outcome measurements are the same in both groups will be required in all cases, and the standard deviation would then be used for both intervention groups. Heterogeneity will be assessed using the *I*^2^ statistic. *I*^2^ ≥ 50% represented high heterogeneity. To detect the impact of each data set on the overall effects of the analyses, sensitivity analysis will be performed by sequentially deleting a single study involved in the meta-analysis. Subgroup analysis will be performed based on the different follow-up periods. Risk ratios (RRs) with a 95% CI were used to assess dichotomous outcomes. The inverse variance and Mantel-Haenszel methods will be used to combine separate statistics. We will evaluate whether asymmetry was due to publication bias or to a relationship between the trial size and effect size using funnel plots. A *P* value <.05 will be considered statistically significant.

### 2.6 Patient and public involvement

No patients will be involved in this study.

## 3 Discussion

The primary objective of this study was to determine whether gait retraining for runners with PFP could be an effective and safe treatment. The result of this study will illustrate whether there is a significant clinical difference in type of retraining program such as step rate, running cadence, time of training sessions, strike pattern, and stride length. As the number of runners and running events has been increasing steadily since the 2000 s,^[24]^ running related injuries were more common. It is thought that altering runners’ technique would help for less running related injuries. This was proved by some biomechanical studies that forefoot landing and step rate increase result in lower cumulative patellofemoral joint stress in healthy runners, with the forefoot landing being the most effective,^[11,25]^ and shortened step length also contribute to less patellofemoral joint stress.^[15]^ Despite of significant difference in biomechanical research, a study published most recently presented clinical benefits, improvement of pain symptoms and functional scores, was not accompanied with significant biomechanics differences that could entirely explain clinical improvement after the 3 gait retraining interventions.^[21]^

There are many treatments aiming to solve PFP in both recreational runners and professional runners, including patient education, exercise therapy targeting both knee and hip muscle strength, and foot orthoses wearing,^[26]^ physical therapy such as patellofemoral joint mobilization, patellar taping, and ultrasound et al.^[27]^ But some review articles indicated a limited effect of these interventions.^[12,28]^ Although previous systematic review has evaluated the value of gait retraining for running injuries, they included only a small number of studies, which were no comparison between some interventions and gait retraining when accompanied with other treatment. As some studies focus on effect of gait retraining for clinical practice exit,^[18–21]^ it is necessary to further evaluate the effect of gait retraining for runners. We aim to use enough studies to ensure adequate power for the meta-analysis. We expect to systematically assess whether gait retraining can give positive effect for runners to relieve pain and improve functions of knee. This study will include the largest amount of studies that systematically assess the efficacy and safety of gait retraining for runners suffered from PFP. The results of this review may help to give available suggestions for runners and to provide reliable evidence for its further application.

## Acknowledgments

We thank American Journal Experts for its linguistic assistance during the preparation of this manuscript.

## Author contributions

**Conceptualization:** Shoubin Lei.

**Data curation:** Huan Xiao.

**Formal analysis:** Zeng Li.

**Investigation:** Huan Xiao, Zeng Li.

**Methodology:** Huan Xiao.

**Software:** Zeng Li.

**Supervision:** Zeng Li.

**Writing – original draft:** Huan Xiao.

**Writing – review & editing:** Shoubin Lei.
